# Early ERP Signature of Hearing Impairment in Visual Rhyme Judgment

**DOI:** 10.3389/fpsyg.2013.00241

**Published:** 2013-05-06

**Authors:** Elisabet Classon, Mary Rudner, Mikael Johansson, Jerker Rönnberg

**Affiliations:** ^1^Linnaeus Centre HEAD, The Swedish Institute for Disability Research, Department of Behavioural Sciences and Learning, Linköping UniversityLinköping, Sweden; ^2^Psychiatric Clinic, The University Hospital in LinköpingLinköping, Sweden; ^3^Department of Psychology, Lund UniversityLund, Sweden

**Keywords:** event-related potentials, hearing impairment, phonology, visual rhyme judgment, inter-stimulus interval, N2, N400, FP

## Abstract

Postlingually acquired hearing impairment (HI) is associated with changes in the representation of sound in semantic long-term memory. An indication of this is the lower performance on visual rhyme judgment tasks in conditions where phonological and orthographic cues mismatch, requiring high reliance on phonological representations. In this study, event-related potentials (ERPs) were used for the first time to investigate the neural correlates of phonological processing in visual rhyme judgments in participants with acquired HI and normal hearing (NH). Rhyme task word pairs rhymed or not and had matching or mismatching orthography. In addition, the inter-stimulus interval (ISI) was manipulated to be either long (800 ms) or short (50 ms). Long ISIs allow for engagement of explicit, top-down processes, while short ISIs limit the involvement of such mechanisms. We hypothesized lower behavioral performance and N400 and N2 deviations in HI in the mismatching rhyme judgment conditions, particularly in short ISI. However, the results showed a different pattern. As expected, behavioral performance in the mismatch conditions was lower in HI than in NH in short ISI, but ERPs did not differ across groups. In contrast, HI performed on a par with NH in long ISI. Further, HI, but not NH, showed an amplified N2-like response in the non-rhyming, orthographically mismatching condition in long ISI. This was also the rhyme condition in which participants in both groups benefited the most from the possibility to engage top-down processes afforded with the longer ISI. Taken together, these results indicate an early ERP signature of HI in this challenging phonological task, likely reflecting use of a compensatory strategy. This strategy is suggested to involve increased reliance on explicit mechanisms such as articulatory recoding and grapheme-to-phoneme conversion.

## Introduction

Over the recent decades there has been a steady aging of the world’s population, a trend that is expected to continue throughout this century (Lutz et al., 2008). Hearing impairment (HI) is tightly linked to age and consequently there will also be a steady increase in the number of people living with a hearing loss. In most cases HI is acquired after the important years in childhood when spoken and written language are established. In these circumstances, phonological abilities have usually had the chance to develop normally, which is not the case when hearing loss is prelingual. However, when auditory functions decline, changes may occur in the neural processing and representation of sounds. For example, several studies have found that acquired HI has a negative effect on phonological awareness as measured by visual rhyme judgment. Such results indicate that the representations of speech sounds in long-term memory, i.e., phonological representations, may gradually deteriorate when hearing is compromised (Lyxell et al., 1994, 1998, 2003; Andersson and Lyxell, 1998; Andersson, 2002; Lee et al., 2007; Lazard et al., 2010, 2011; Rönnberg et al., 2011).

However, only two brain imaging studies have investigated the neural correlates of visual rhyme judgment in deafened adults (Lazard et al., 2010, 2012). Both used functional Magnetic Resonance Imaging (fMRI) to examine whether preoperative phonological processing could predict outcome of cochlear implantation. Results showed that the hearing-impaired participants either relied on the same, dorsal phonological route as the participants with normal hearing (NH), or they activated a ventral temporo-frontal route, including increased activation in anterior, triangular, parts of the left inferior frontal gyrus (IFG). This area has been linked to semantic access, while the opercular part of the left IFG has been associated with phonological manipulation such as grapheme-to-phoneme conversion and subvocal rehearsal (Jobard et al., 2003; Vigneau et al., 2006; Aparicio et al., 2007). The authors suggested that activation of the ventral route reflected a more global semantic strategy, in spite of the phonological demands inherent in the task (Lazard et al., 2010). In addition, the right posterior superior temporal gyrus/supramarginal gyrus (PSTG/SMG), an area normally involved in processing of environmental sounds, was activated significantly more in the hearing-impaired participants compared to the participants with NH (Lazard et al., 2010). The right temporal recruitment increased with duration of hearing loss and showed a negative correlation with rhyme judgment performance. These results were interpreted to indicate a functional reorganization in response to auditory deprivation. With reference to studies showing that cross-modal plasticity in auditory speech areas of the brain can lead to an enhanced receptiveness to visual linguistic input following acquired hearing loss (Lee et al., 2007; Champoux et al., 2009), the authors suggested that right lateralized temporal areas are recruited for phonological processing as left-lateralized temporal regions become more responsive to visual information (Lazard et al., 2012).

Hence, both behavioral and fMRI data indicate certain long-term changes in phonological processes following postlingual hearing loss, changes that are related to representations of sound stored in semantic long-term memory and neural plasticity rather than the on-line distortion of auditory input *per se*. The purpose of the present study was to take advantage of a method with high temporal resolution, event-related potentials (ERPs), to examine the time-course of phonological processing in visual rhyme judgment of individuals with acquired HI.

Event-related potentials have proven to be an important tool in the study of language processes. The N400, a centroparietal negativity peaking at around 400 ms after stimulus onset, is perhaps the most intensely studied language related ERP component (Barber and Kutas, 2007; Kutas and Federmeier, 2011). Stimuli that are incongruent in a particular context elicit larger (more negative) N400 amplitudes than congruent stimuli. The N400 is contingent upon both the stored representation itself and the retrieval cues provided by the context and its amplitude is enhanced whenever extra allocation of resources is required for access and integration (Barber and Kutas, 2007; Lau et al., 2008). A number of studies have investigated phonological processing by examining ERPs elicited during visual rhyme judgments. The N400 is consistently amplified in non-rhyming as compared to rhyming conditions (Polich et al., 1983; Rugg, 1984a,b; Kramer and Donchin, 1987; Rugg and Barrett, 1987; Weber-Fox et al., 2003; Khateb et al., 2007, 2010). This phonological N400 has been interpreted as a marker of mismatch between the phonology of a presented stimulus and that of the expected/selected candidates momentarily available in short-term memory (Khateb et al., 2007).

A few ERP studies have manipulated both phonology and orthography by using word pairs that rhyme (R+) or not (R−) and are orthographically similar (O+) or dissimilar (O−) (Polich et al., 1983; Kramer and Donchin, 1987; Rugg and Barrett, 1987; Weber-Fox et al., 2003). The visual, orthographic, cues either match (R+O+, R−O−) or mismatch (R+O−, R−O+) the phonological cues. In participants without communicative disabilities the effect of phonological and orthographic mismatch is additive: the N400 is consistently largest in response to non-rhyming, orthographically dissimilar word pairs (R−O−, e.g., shirt – witch), intermediate in the mismatching conditions (R+O−, e.g., moose – juice, R−O+, e.g., some – home), and smallest in response to rhyming word pairs that are orthographically similar (R+O+, e.g., load – toad). Consequently, the component can be used as an index of normal brain functioning against which specific populations with phonological processing deficits can be compared. For instance, N400 attenuation and onset delay suggest reduced processing efficiency and slower processing, respectively, and both have been found in individuals with dyslexia (Ackerman et al., 1994; McPherson et al., 1996, 1998). To our knowledge it has not previously been investigated whether acquired long-term severe HI impacts the phonological N400.

Earlier ERP markers of orthographic and phonological processing in silent reading paradigms, the N2-negativities, appear at around 200 ms after stimulus onset. For example, studies have found enhanced N2-like responses to homophonically related word pairs (e.g., plain – plane; Niznikiewicz and Squires, 1996), presentation of syllables that violate expectations (e.g., judging whether the syllable “com” was included or not in the expected word “botte”; Proverbio and Zani, 2003), words orthographically but not phonologically incongruent to expected words (e.g., The ship disappeared into the thick phog [fog]; Newman and Connolly, 2004; Vissers et al., 2006), words violating phonological expectancy (e.g., Rob looked at his watch to check the skull [thyme, tyme, or time]; Savill et al., 2011) and to pseudowords as compared to unpronounceable letter strings and real words (Simon et al., 2004; Martin et al., 2006). These negativities have been interpreted as reflecting detection of conflict between outputs of orthographic and phonological analyses (Niznikiewicz and Squires, 1996), phonologically based lexical retrieval (Simon et al., 2004), or violation of orthographic form expectations (Newman and Connolly, 2004).

Event-related potentials effects in this early time-window have hitherto not been reported in visual rhyme judgment studies. It needs to be noted that few of these studies report whether early time-windows, i.e., before around 300 ms after stimulus onset, have been analyzed. However, the sensitivity of N2-like negativities to conflicts between phonology and orthography renders them interesting in relation to visual rhyme judgments made by individuals with HI. Typically, HI has no impact on performance when the visual cues provided by the orthography aid rhyme judgment. It is specifically when the phonological and orthographic cues conflict, for example when words look alike but don’t rhyme (e.g., some – home), that individuals with HI do significantly worse than individuals with NH.

Sentences that are syntactically incorrect elicit a late positivity, the P600 (for reviews, see Kuperberg, 2007; Van Petten and Luka, 2012). In language studies, late positive-going modulations are generally thought to reflect a continued analysis or reanalysis following violations of linguistic predictions or rules (Federmeier et al., 2007; Kuperberg, 2007; van de Meerendonk et al., 2010). Although mainly studied in relation to syntactic processing, the P600 can also be elicited by orthography/phonology conflicts. For instance, Vissers et al. (2006) found an enhanced P600 in response to pseudohomophones of highly predictable words (e.g., In that library the pupils borrow bouks [books]). Liu et al. (2011) used lines from well-known Chinese poems in which one word had been substituted with either a synonym, i.e., a word semantically congruent but phonologically incongruent to the expected word, or a homophone, i.e., a word semantically incongruent but phonologically congruent to the expected and found larger P600 in the synonym condition. In the study by Savill and Thierry (2011) the P600 differentiated between homophones and non-homophones (e.g., horce-horse versus horle-horce) in controls but not in dyslexics.

As is the case with the early negativities, late positivities are typically not reported in rhyme judgment studies. An exception is an early study by Rugg (1984a) in which a late positive component (referred to as LPC) was found to be sensitive to phonological congruency in terms of its peak latency. The latency was significantly shorter in response to rhymes than non-rhymes in words and non-words, an effect the author interpreted as an index of the extra time required to evaluate and categorize phonologically incongruent stimuli. Thus, any hearing related difficulties in the rhyme judgment task could also be reflected by deviations in the late positivities.

The work of Neely and colleagues (Neely, 1977; Neely et al., 1989) has established that contextual cues can facilitate access to stored lexical representations via both implicit and explicit processes. When <500 ms separates the onsets of a prime and a target word, facilitation is predominantly mediated by implicit spreading of activation in the neuronal semantic long-term memory network. Longer latencies between prime and target onsets allow for involvement of explicit expectancy strategies (Neely, 1977; Neely et al., 1989; Baum and Leonard, 1999; McQueen and Sereno, 2005). Sets of potential targets with a task-relevant relatedness to the prime are generated. When the presented target is consistent with the prime, demands on strategic lexical search and access processes are reduced. However, when the target is inconsistent with the prime, the expectancy set needs to be inhibited and attention shifted to the presented target (Neely, 1977; Lau et al., 2008).

In recent years, a growing body of research has pinpointed the important role of top-down influences in language processing when audition is compromised. For example, access to explicit resources in the form of good working memory capacity is associated with better speech recognition under challenging listening conditions (Lunner, 2003; Gatehouse et al., 2006; Foo et al., 2007; Lunner and Sundewall-Thorén, 2007; Rudner et al., 2011) and supports performance in phonologically demanding tasks (Classon et al., 2013) in individuals with HI.

With respect to the present study, the poorer quality phonological representations associated with HI are likely to interfere with implicit spreading of activation to phonologically related lexical representations. Engagement of explicit top-down processes might then provide an important compensatory pathway to lexical access (Rönnberg et al., 2008). If so, markers of sublexical phonological processes and phonologically mediated lexical access should deviate more under conditions where implicit priming is the dominant process as compared to conditions allowing explicit mechanisms to kick in (Rönnberg et al., 2010).

The aim of the present study was to investigate the effects of HI on the ERP correlates of phonological processing. Participants with postlingually acquired moderate-to-severe HI for at least 10 years and a NH control group performed a visually presented rhyme judgment task in four conditions: two matching (R+O+ and R−O−) and two mismatching (R+O− and R−O+). The inter-stimulus interval (ISI) was either short (50 ms, i.e., 250 ms between word onsets) or long (800 ms, i.e., 1000 ms between word onsets) to investigate if behavioral performance and/or ERP correlates differed as a function of the possibility to engage top-down, explicit processes. These ISI’s were chosen because they represent intervals frequently studied in the literature on implicit/explicit facilitation (e.g., Neely, 1977; McQueen and Sereno, 2005; Lau et al., 2008).

We hypothesized that the neural response of participants with HI would be different from that of NH participants in N400 amplitude and/or peak latency in the mismatching rhyme task conditions. In addition we expected any N400 difference between the groups to be larger in short ISI than in long ISI because the latter leaves more room for engagement of compensatory processes. We also expected HI to be associated with a larger sensitivity to conflicts between orthographic and phonological cues as reflected by enhanced N2-like responses in the mismatching rhyme conditions. Lastly, we chose to analyze a time-window following the N400 to capture any late positivities sensitive to hearing status.

## Materials and Methods

The data reported below were collected as part of a larger study which included a test battery to assess auditory and cognitive function and an off-line episodic recognition memory test. The primary focus of this article is the behavioral and ERP data collected in a rhyme judgment task.

### Participants

Fifty-three adults participated in this study. They were all native Swedish speakers and reported normal or corrected to normal vision, no psychiatric or neurological disorders, and no history of reading disability. All were right-handed except for one participant with NH. Twenty-six individuals (12 women) with a mean age of 63 years (SD = 6.50) were recruited from the audiological clinic at Linköping University Hospital. All had postlingually acquired moderate-to-severe sensorineural hearing loss (HI). Mean best ear pure tone average measured across 500, 1000, 2000, and 4000 Hz (BestEarPTA) was 72.27 (SD = 10.12, range 59–109). The mean duration of hearing loss was 36 years (SD = 12.07, range 10–61) and mean age at hearing loss onset was 26 years (SD = 13.32, range 4–53). Duration and age of onset were self-reported responses to the question when they first became aware of having a hearing problem. All were bilaterally fitted with hearing aids and the average length of time since fitting was 19 years (SD = 11.50, range 3–47).

Twenty-seven (11 women) individuals with NH, here defined as a BestEarPTA of <26 dB hearing loss, were recruited from the general population and constituted the reference group (NH). Their mean BestEarPTA was 14.42 (SD = 6.52, range 3–25) and mean age 62 (SD = 8.22). There was no difference between the two groups in age.

The study was approved by the regional ethics committee in Linköping (Dnr 77-09) and all participants provided written informed consent before testing started.

### General procedure

Participants were tested at two separate occasions. In the first session, background data were collected and the participant took part in the ERP experiment. After this, an episodic recognition memory task and the test battery assessing cognitive abilities were administered. The test battery included tests of word comprehension (“Ordförståelse B,” Word comprehension B; Järpsten, 2002), phonological short-term memory (digit span forward), working memory capacity (Reading span; Rönnberg et al., 1989), non-word reading (“Ljuden ger orden,” The sounds give the word; Lundberg and Wolff, 2003), orthographic word-pseudohomophone discrimination (“Bokstäverna ger ordet,” The letters give the words; Lundberg and Wolff, 2003) and semantic and phonemic verbal retrieval (category and letter fluency; Benton, 1968). These tests are described in detail elsewhere (Classon et al., 2013, under revision). Total length of first the session was approximately 4 h, including time for applying and removing the ERP net and 30 min break after the recognition task. The rhyme task took around 40 min to complete. In a second session an experienced audiologist collected audiograms and speech reception thresholds (using Hagerman sentences; Hagerman and Kinnefors, 1995) from all participants.

### Experimental materials, rhyme judgment task

Rhyme task stimuli consisted of 192 word pairs of which 48 were orthographically similar rhymes (R+O+, *korp – torp*, [kår:p] – [tår:p]), 48 orthographically dissimilar rhymes (R+O−, *helg – välj*, [hel:j] – [vel:j]), 48 orthographically similar non- rhymes (R−O+, *sant – saft*, [san:t] – [saf:t]) and 48 orthographically dissimilar non- rhymes (R−O−, *bröd – spik*, [brö:d] – [spi:k]). Grapheme-to-phoneme correspondence is more consistent in Swedish than, for example, English (Seymour et al., 2003) and few orthographically dissimilar Swedish words rhyme. Therefore R+O− word pairs included low-frequency words and words from different open word classes (nouns, verbs, adjectives, and adverbs). Consequently, words for the other conditions were selected to match the R+O− words. Further, specific letter combinations can typically only be pronounced in one way in Swedish. Swedish R−O+ word pairs thus have orthographically similar, but not identical, final syllables. Different strategies were adopted to create similar word-end gestalts: in most cases word-endings differed by one letter (e.g., sant – saft, [san:t] – [saf:t]), in other cases letters were rearranged (e.g., rost – fots, [rås:t] – [fo:ts]), or added (e.g., besked-beskydd, [be∫ẹ:d] [be∫ỵd:]). The variety of solutions reduced the predictability of the orthographic form of the second word in each pair and ensured the saliency of mismatch in the R−O+ condition. All rhyme task words were three to nine letters long, mono- or disyllabic and selected from the Swedish text corpus PAROLE (Språkbanken, University of Gothenburg)^1^. The distribution of mono- and disyllabic words, word classes and stress patterns was even over conditions and positions (prime word, target word) in word pairs. MANOVA with word length and word frequency as dependent variables, and condition (four levels: R+O+, R+O−, R−O+, R−O−) and position (two levels: prime, target) as independent variables, showed that there were no differences in either word frequency or word length over conditions or position (see Table 1 for descriptive statistics). The 192-rhyme task word pairs were pseudorandomized into a single list. No more than five “yes” (R+O+ and R+O−) or “no” (R−O+ and R−O−) answers, and no more than three consecutive trials from the same condition was allowed.

**Table 1:** **Descriptive statistics of the word stimuli divided by condition**.

Condition	Word length			Word frequency		
	*M*	SD	Range	*M*	SD	Range
R+O+	4.97	1.44	6	381.23	762.91	5259
R+O−	4.93	1.22	5	400.57	1075.00	8582
R−O+	4.85	1.22	6	377.94	1028.00	6521
R−O−	5.01	1.32	5	382.61	1160.10	8448

### Procedure

#### Rhyme judgment task

**Figure 1:**
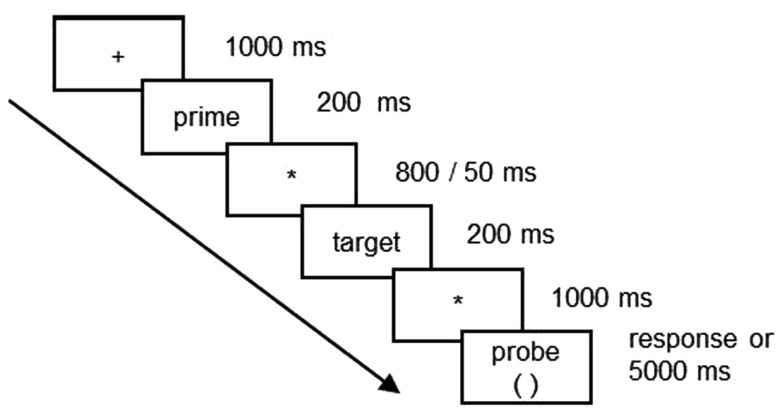
**The ERP paradigm**.

E-prime software (version 2.0)^2^ from Psychological Software Tools, Inc., was used for stimulus presentation and collection of behavioral responses. Words were presented centrally on a 17″ liquid crystal display computer screen in white lowercase letters against a black background. The horizontal visual angle of the word stimuli were 2.3–5.3°. Each trial began with a fixation cross displayed for 1000 ms. The fixation cross was followed by a 200-ms presentation of the prime word. An ISI of either 50 or 800 ms preceded presentation of the target word. The target word remained on screen for 200 ms and 1000 ms after target word offset, a response probe appeared (see Figure 1). The task was to decide with a button press whether the words in each paired rhymed or not and from probe onset participants had 5 s to respond. In order to encourage a phonological strategy, participants were instructed not to pay attention to how words were spelled, but decide solely on basis of word pronunciation. The rhyme judgment list was presented twice to all participants, once with short ISI and once with long ISI. Order of presentation was counterbalanced.

#### ERP recording and analyses

The EGI (Electrical Geodesics Inc., Eugene, OR, USA) Geodesics Net Amps system with 128-channel, Ag/AgCl electrode, HydroCel Geodesic Sensor Nets, a Net Amps 300 Amplifier and NetStation software version 4.4.2 was used to acquire and analyze EEG data. The EEG was continuously recorded at a 250-Hz sampling rate and electrode impedances were kept below 50 kΩ. A vertex reference was used during acquisition. Offline, the EEG was 0.30–30 Hz bandpass filtered. The continuous EEG was segmented into epochs from 200 ms before either target onset (long ISI), or prime onset (short ISI), to 1000 ms post target onset. The longer epochs extracted in the short ISI ensured inclusion of a time-window for baseline correction that was free from ERPs elicited by the prime. Only trials with correct answers were retained for statistical analyses. The NetStation Artifact Detection tool was then used to detect artifacts. Thresholds were set to ±100 μV for eye blinks and ±45 μV for horizontal eye-movements. EEG channel thresholds were set to voltage changes larger than ±70 μV within 150 ms intervals, or <1 μV within a segment. A channel was marked bad for the entire session if it was deemed bad in more than 20% of the trials. Segments containing more than 10 bad channels, eye-movements or eye blinks were rejected. The Bad Channel replacement tool was applied to interpolate bad channels in good segments, about 1.5% of the data, using spherical splines. All data were also visually inspected. Accepted segments were averaged in each condition, re-referenced to the average reference, and baseline corrected either over the 100-ms before target onset (long ISI), or over the 100-ms before prime onset (short ISI). Overall, 15% of segments, evenly distributed over the conditions, from correctly answered trials were excluded. The average number of artifact-free segments from correctly answered trials in each condition is displayed in Table 2.

**Table 2:** **Mean number of segments, divided by condition and inter-stimulus interval (ISI), that were retained for analyses after exclusion of trials with incorrect answers and artifact contaminated segments**.

Condition	Long ISI (800 ms)			Short ISI (50 ms)		
	*M*	SD	Range	*M*	SD	Range
R+O+	39	6.85	20–47	38	6.81	22–47
R+O−	32	8.15	16–45	32	7.08	17–45
R−O+	36	8.33	19–47	34	9.15	17–45
R−O−	41	6.50	20–48	40	7.01	19–48

**Figure 2:**
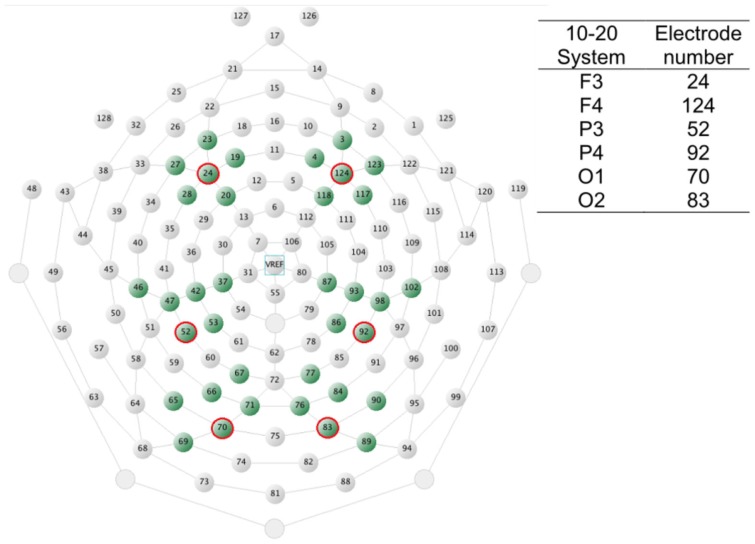
**A schematic view of the HydroCel Geodesic Sensor Net as seen from above with the nose up and neck down**. The electrodes chosen for statistics extraction are shaded. The inserted table shows corresponding 20–10 system electrodes.

Six groups of electrodes representing frontal, centroparietal, and occipital areas in both hemispheres were chosen for statistical analyses based on previous literature and visual inspection of the waveforms. The electrode groups are displayed in Figure 2 together with a table showing corresponding electrode numbers based on the 10–20 international system (for a more comprehensive table of HydroCel Geodesic Sensor Nets – 10–20 system electrode number correspondences, see Luu and Ferree, 2000). Three consecutive time-windows were used for analyses: 100–300, 300–500, and 500–700 ms post target word onset. Mean amplitude (mean voltage value in a time-window) and peak latency (time in ms from target word onset to the maximum amplitude point in a time-window) were extracted for each electrode in the electrode groups. In case the peak latency was recorded at, or close to, either borders of a time-window, waveforms were visually inspected to verify that the statistically identified peak was not part of a deflection whose maximum amplitude fell outside the time-window. If it was, the latency to the second-to-maximum peak was manually extracted. For each participant, time-window and rhyme task condition the resulting mean amplitude and peak latency values were then individually averaged across the six electrodes in each group.

#### Statistical analyses

Event-related potential mean amplitudes were analyzed by separate mixed ANOVAs conducted in each time-window (100–300, 300–500, and 500–700 ms) and ISI (long, short). Rhyme (two levels: R+, R−), orthography (two levels: O+, O−), antpos (three levels: frontal, centroparietal, occipital), and hemisphere (two levels: left, right) were entered as within-participants variables and group (two levels: NH, HI) as between-participants variable. Peak latencies of the components of interest were analyzed by mixed ANOVAs with the factors rhyme, orthography, and group in the topographic regions where the components had been identified as maximal by the omnibus ANOVA (e.g., right centroparietal). Separate follow-up ANOVAs for each group were conducted to verify presence of the N400. Greenhouse–Geisser correction was applied whenever Mauchly’s test of sphericity was significant. Interactions were examined by pairwise *t*-tests with Bonferroni correction for multiple comparisons and effect sizes were computed for the pairwise comparisons. The effect sizes were further used to help interpretation of the data, i.e., as indicators of which topographic region was most sensitive to differences between conditions. Behavioral data (percent correct responses) were analyzed with separate three-way (rhyme × orthography × group) mixed ANOVAs for each ISI.

## Results

### Cognitive tests

Group differences in performance were found on only one of the cognitive tests (Table 3). NH and HI were comparable on working memory capacity, phonological short-term memory, verbal ability, semantic verbal retrieval, phonological decoding, and orthographic decoding [*ts*(51) ≤ 0.44, *ps* ≥ 0.07, ns]. However, HI were outperformed by NH in phonemically based verbal retrieval as measured by the letter fluency task [*t*(51) = 2.55, *p* = 0.014].

**Table 3:** **Means and standard deviations for the normal hearing (NH) and hearing-impaired (HI) groups on the cognitive tests**.

Test	NH		HI	
	*M*	SD	*M*	SD
Reading span	41.52	8.88	39.46	10.14
Word comprehension	30.96	1.58	29.85	2.87
Non-word reading	28.22	9.00	25.23	6.90
Orthographic word-pseudohomophone discrimination	51.89	12.44	44.92	18.57
Letter fluency	30.85*	8.29	25.23*	7.73
Category fluency	56.52	11.86	51.23	8.76
Digit span forward	58.89	10.01	55.81	10.59

*p < 0.05.

### Rhyme judgment: Behavioral results

#### Long ISI

**Figure 3:**
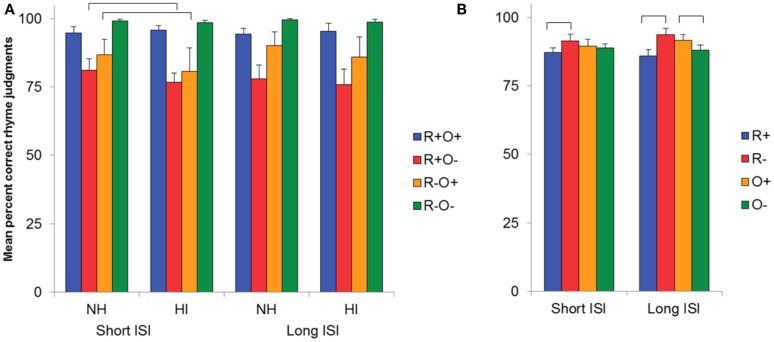
**(A)** Mean percent correct rhyme judgments as a function of hearing status and condition in short and long ISI. **(B)** Mean percent correct rhyme judgments collapsed over the rhyming (R+), non-rhyming (R−), orthographically similar (O+) and orthographically dissimilar (O−) conditions, all participants, in short and long ISI. Error bars represent 95% CI.

ANOVA resulted in main effects of both rhyme [*F*(1, 51) = 18.67, MSE = 168.62, *p* < 0.001, *η*^2^ = 0.27] and orthography [*F*(1, 51) = 6.11, MSE = 105.10, *p* = 0.017,*η*^2^ = 0.11] in long ISI. More errors were made in response to rhyming word pairs than to non-rhyming word pairs and to orthographically dissimilar word pairs than to orthographically similar word pairs. There was also a rhyme × orthography interaction [*F*(1, 51) = 163.69, MSE = 68.50, *p* < 0.001, *η*^2^ = 0.76]. As can be seen in Figure 3, more errors were made in the mismatching (R+O−, R−O+) than in the matching (R+O+, R−O−) conditions (*p*s < 0.001, *r*s > 0.69). Out of the two mismatching conditions, performance was lowest in R+O− [*t*(51) = 6.96, *p* < 0.001, *r* = 0.70]. There were no group differences.

#### Short ISI

A similar pattern of results, with more errors in the mismatching than the matching conditions and more errors in R+O− than in R−O+, was found in short ISI [main effect of rhyme, *F*(1, 51) = 6.50, MSE = 139.16, *p* = 0.014, *η*^2^ = 0.11, and rhyme × orthography interaction, *F*(1, 51) = 154.09, MSE = 84.30, *p* < 0.001, *η*^2^ = 0.75, with *p*s < 0.001 and *r*s > 0.46 in *post hoc* comparisons]. Further, performance differed between the two groups: HI made significantly more errors than NH in both mismatching conditions [rhyme × orthography × group interaction, *F*(1, 51) = 4.73, MSE = 84.93, *p* = 0.034, *η*^2^ = 0.08, *t*_R+O−_(51) = 2.51, *p* = 0.015, *r* = 0.33, and *t*_R − O+_(51) = 3.42, *p* = 0.001, *r* = 0.44 in *post hoc* comparisons].

The finding of lower performance by HI than NH in the mismatching conditions in short ISI, contrasting with similar performance in long ISI, indicates that HI could engage top-down processes, consuming more time than allowed by short ISI, to overcome phonological difficulties. The pattern of data (see Figure 3) further suggests it was primarily R−O+ performance that improved with the opportunity to employ explicit processing. In order to analyze benefit from long ISI, an index of change was computed by subtracting performance in short ISI from performance in long ISI in each condition. Results of one-sample *t*-tests showed significant improvement in R−O+ only, and this was the case for both groups. HI performance improved with 5% and that of NH by 3.5% [*t*_NH_(26) = 2.49, *p* = 0.019, *r* = 0.44 and, *t*_HI_(25) = 2.24, *p* = 0.034, *r* = 0.41] but there was no difference between the groups.

### Rhyme judgment: ERP results

**Figure 4:**
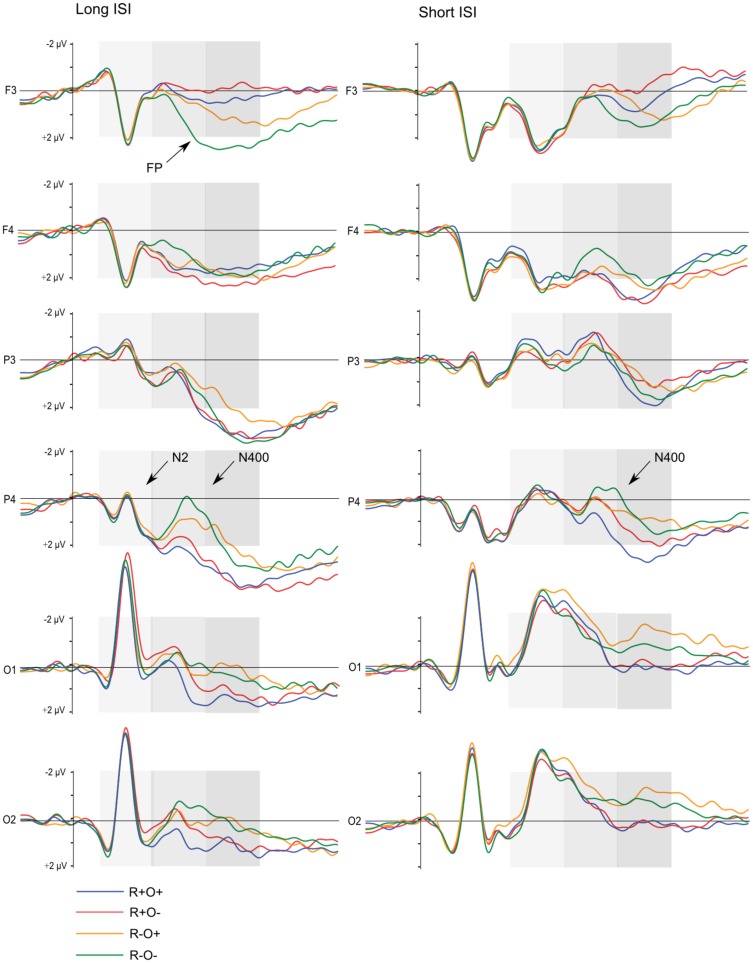
**Grand average ERP waveforms elicited by R+O+, R+O−, R−O+, and R−O− target words in long and short ISI, all participants**. The shaded areas mark the 100–300, 300–500, and 500–700 ms time-windows, respectively.

**Figure 5:**
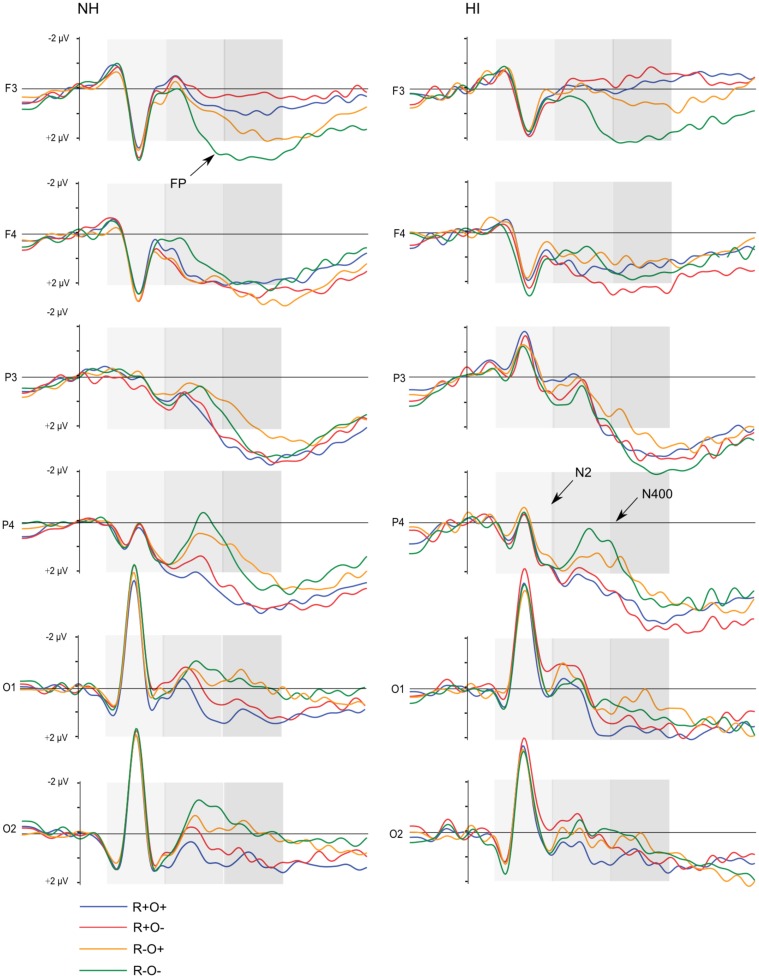
**Grand average ERP waveforms elicited by R+O+, R+O−, R−O+, and R−O− target words in each group, long ISI condition**. The shaded areas mark the 100–300, 300–500, and 500–700 ms time-windows, respectively.

**Figure 6:**
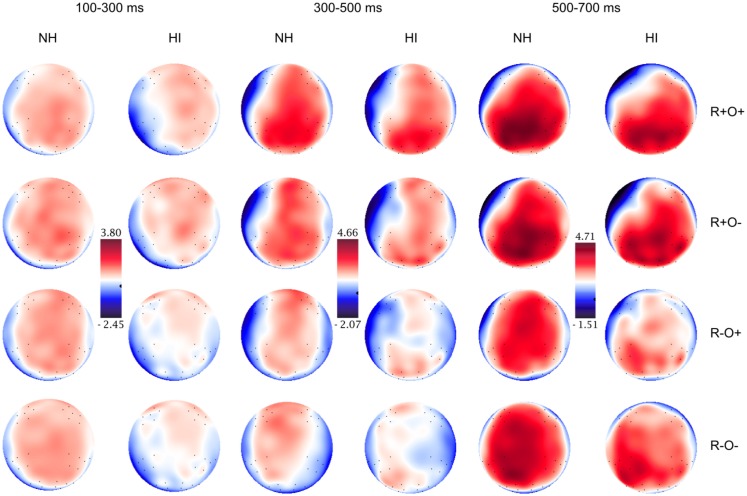
**Topographical distribution of the grand averaged ERPs in the normal hearing (NH) and hearing-impaired (HI) groups in the 100–300, 300–500, and 500–700 ms time-windows, long ISI**. The rows show the distribution over the R+O+, R+O−, R−O+, and R−O− rhyme task conditions.

**Figure 7:**
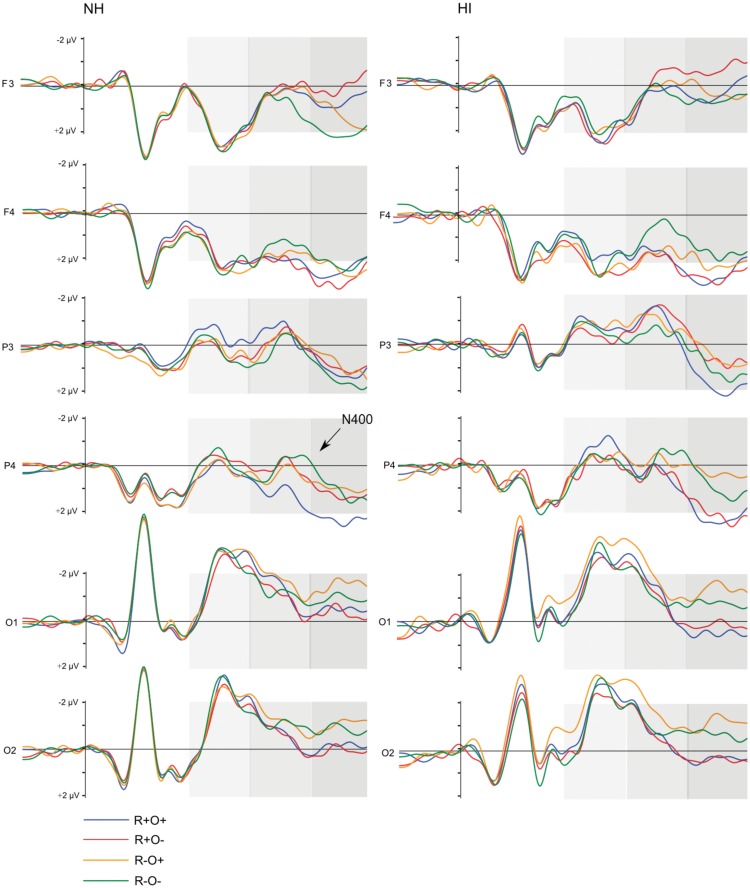
**Grand average ERP waveforms elicited by R+O+, R+O−, R−O+, and R−O− target words in each group, short ISI condition**. The shaded areas mark the 100–300, 300–500, and 500–700 ms time-windows, respectively.

**Figure 8:**
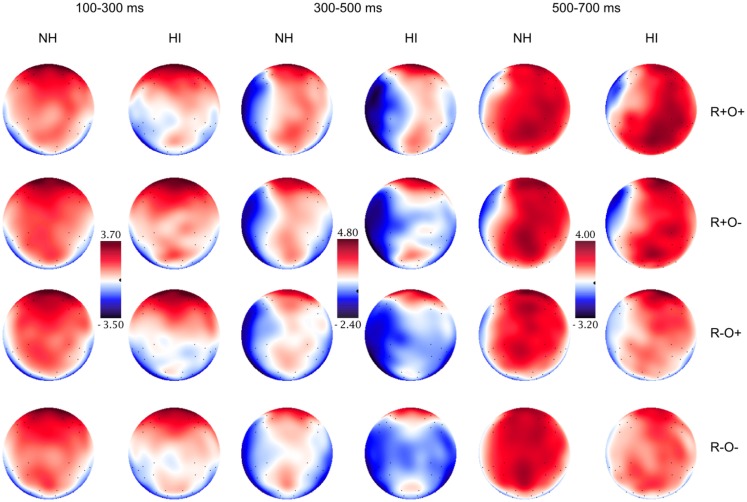
**Topographical distribution of the grand averaged ERPs in the normal hearing (NH) and hearing-impaired (HI) groups in the 100–300, 300–500, and 500–700 ms time-windows, short ISI**. The rows show the distribution over the R+O+, R+O−, R−O+, and R−O− rhyme task conditions.

Grand average waveforms for all participants in long and short ISI are displayed in Figure 4. Figures 5 and 6 show the grand average waveforms and their topographical distribution for each group in long ISI, Figures 7 and 8 in short ISI. Overall, waveforms showed an N170 at occipital sites, an early negativity followed by a distinct N400 over the right centroparietal area and a left-lateralized frontal positivity (FP). Results of the ANOVA analyses including all participants are summarized in Table 4. Whenever electrodes have a corresponding 10–20 system label, that label is used below; otherwise the electrodes are labeled according to their HydroCel Geodesic Sensor Net number.

**Table 4:** **Statistics for all effects of the ANOVAs conducted on mean amplitudes in the three time-windows analyzed**.

Condition	Effect	Time-window					
		100–300 ms		300–500 ms		500–700 ms	
		*F*	*p*	*F*	*p*	*F*	*p*
Long ISI	R	0.14	ns	35.60	<0.001	7.30	<0.01
	O	2.69	ns	2.63	ns	11.07	<0.01
	A	14.77	<0.001	1.61	ns	15.23	<0.001^a^
	H	18.64	<0.001	12.63	0.001	3.74	ns
	R × O	0.76	ns	6.00	<0.05	9.53	<0.001
	R × A	0.33	ns	25.74	<0.001^a^	50.90	<0.001^a^
	O × A	5.66	<0.05^a^	7.23	<0.01^a^	0.55	ns
	R × O × A	1.63	ns	0.04	ns	0.62	ns
	R × H	2.71	ns	55.50	<0.001	36.41	<0.001
	O × H	0.06	ns	22.88	<0.001	1.34	ns
	R × O × H	1.66	ns	16.26	<0.001	22.49	<0.001
	A × H	3.37	<0.05	10.69	<0.001	15.94	<0.001
	R × A × H	0.02	ns	25.36	<0.001	24.12	<0.001
	O × A × H	4.60	<0.05	11.84	<0.001	4.90	<0.01
	R × O × A × H	3.41	<0.05	17.60	<0.001	13.42	<0.001
Short ISI	R	1.83	ns	17.50	<0.001	17.17	<0.001
	O	3.19	ns	1.92	ns	0.66	ns
	A	50.51	<0.001^a^	9.49	<0.01^a^	10.64	0.001^a^
	H	2.21	ns	52.83	<0.001	39.91	<0.001
	R × O	4.16	<0.05	4.95	<0.05	21.85	<0.001
	R × A	0.37	ns	1.02	ns	30.12	<0.001^a^
	O × A	1.09	ns	2.26	ns	0.62	ns
	R × O × A	1.25	ns	0.58	ns	2.63	ns
	R × H	0.31	ns	54.35	<0.001	49.14	<0.001
	O × H	4.62	<0.05	24.60	<0.001	0.66	ns
	R × O × H	1.71	ns	3.60	ns	10.90	<0.01
	A × H	1.71	ns	7.27	0.001	27.08	<0.001^a^
	R × A × H	1.86	ns	5.59	<0.01	23.39	<0.001
	O × A × H	0.04	ns	0.96	ns	0.06	ns
	R × O × A × H	4.50	<0.05	2.72	ns	5.81	<0.01

*R, rhyme; O, orthography; A, antpos; H, hemisphere*.

*^a^Greenhouse–Geisser correction*.

#### 100–300 ms latency window

##### Long ISI

**Figure 9:**
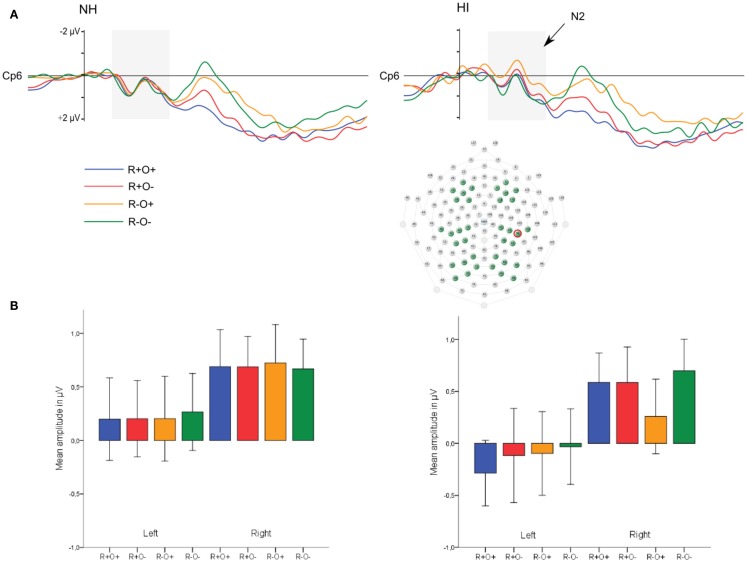
**(A)** Grand average ERP waveforms at electrode Cp6 and **(B)** mean amplitudes in the right hemisphere elicited by R+O+, R+O−, R−O+, and R−O− target words in the 100- to 300-ms latency window, long ISI, divided by group. Error bars represent 95% CI.

In the 100- to 300-ms time-window, ERPs showed an N2-like negativity that differed between conditions over the right centroparietal area (rhyme × orthography × antpos × hemisphere interaction). Its amplitude was significantly larger (more negative) as elicited by R−O+ than the other conditions (mean amplitudes across conditions: 0.65–0.94 μV, SD: 1.02–1.27, *p*s < 0.001, *r*s = 0.46–0.54) while it was not sensitive to condition in terms of peak latency (mean peak latencies over conditions: 164–178 ms, SD: 31.64–36.50). The N2-like negativity was largest at P4 with a mean R−O+ amplitude of −0.31 μV (as compared to R+O+, −0.02; R+O−, −0.16; R−O−, −0.08). Interestingly, the N2-like deflection in R−O+ was driven by HI [rhyme × orthography × hemisphere × group interaction, *F*(1, 50) = 4.58, MSE = 2.09, *p* = 0.037]. As displayed in Figure 9, amplitudes were significantly more negative in R−O+ than in R−O− over the right hemisphere for HI [*t*(50) = 4.60, *p* < 0.001, *r* = 0.42]. The absolute amplitude difference between the conditions was largest in the centroparietal electrode group, where it was most pronounced in electrodes Cp6 (*M*_R−O+_ = 0.11 μV, *M*_R−O−_ = 0.77 μV, see Figure 9) and 86. By contrast, ERPs did not differentiate between conditions in NH. There were no group differences in right centroparietal peak latency [*F*s(1, 50) < 0.715, ns].

##### Short ISI

In short ISI, *post hoc* investigation of the highest order interaction (rhyme × orthography × antpos × hemisphere), focusing on the N2-like negativity described above, showed that, although there was a similar, but more protracted, right centroparietal negative deflection in short SOA, amplitudes were not sensitive to condition (mean amplitudes across conditions: 0.00–0.11 μV, SD: 1.72–1.86; *p*s > 0.191, ns). Neither were right centroparietal peak latencies [mean peak latencies across conditions: 167–175 ms, SD: 44.28–49.08; *F*s(1, 50) < 1.30, ns].

These results were in support of our hypothesis that HI is associated with enhanced N2-like responses. The effect was seen only in long ISI, that is, where the behavioral performance of HI was on a par with that of NH.

#### 300–500 ms latency window

##### Long ISI

As expected, there was a distinct N400 over the right centroparietal area in this time- window (rhyme × orthography × antpos × hemisphere interaction). The N400 was significantly larger (more negative) the more incongruent the condition: R−O− > R−O+ > R+O− > R+O+ (mean amplitudes across conditions: 0.50–2.17 μV, SD: 1.40–1.57, *p*s < 0.001, *r*s = 0.55–0.81). The amplitude differences between R−O− and R+O+ was largest at electrode Cp4 with mean amplitudes of −0.06 and 1.98 μV respectively. Peak latencies were significantly shorter for rhymes than non-rhymes [*M*_Rhymes_ = 371 ms, SD = 40.24, *M*_Non-rhymes_ = 394 ms, SD = 35.33, *F*(1, 51) = 26.97, MSE = 1060.30, *p* < 0.001, *η*^2^ = 0.35]. The N400 continued over bilateral occipital sites, but here it did not differ between the two non-rhyming conditions, such that R−O− = R−O+ > R+O− > R+O+ (*p*s < 0.001, *r*s = 0.49–0.75).

Follow-up ANOVAs showed that the N400 was sensitive to rhyme and orthography in both groups with more negative amplitudes in response to non-rhyming and orthographically dissimilar word pairs, respectively (main effects of rhyme and orthography, *p*s < 0.035, *η*^2^ = 0.28–0.68). Both groups also showed longer N400 latencies in the non-rhyming conditions [main effect of rhyme, *F*_NH_(1, 26) = 15.62, MSE = 1131.96, *p* = 0.001, *η*^2^ = 0.38; *F*_HI_(1, 24) = 10.84, MSE = 1011.90, *p* < 0.003, *η*^2^ = 0.31; mean peak latencies across conditions: 366–403 ms, SD: 37.27–54.52]. However, there were no group differences in either N400 amplitudes or peak latencies [*F*s(1, 50) < 1.25, ns].

In the left frontal electrode group, ERPs showed a different pattern. As can be seen in Figures 4 and 5, an extended positivity in response to the non-rhyming conditions, particularly R−O−, starts differentiating between conditions from around 400 ms such that R+O+ = R+O− < R−O+ < R−O− (*p*s < 0.002, *r*s = 0.41–0.92).

##### Short ISI

In short ISI, an N400 effect differentiated between rhymes and non-rhymes over all right hemisphere sites (rhyme × antpos × hemisphere interaction, *p*s < 0.001, *r*s = 0.62–0.69). The largest effect size and mean amplitude difference was measured in the centroparietal area (*M*_Rhyme_ = 0.88 μV, SD = 1.90, *M*_Non-rhyme_ = 0.15 μV, SD = 1.79). Right centroparietal N400 peak latencies were also sensitive to condition, being longer in response to non-rhyming than rhyming, and orthographically dissimilar than similar target words [main effect of rhyme, *F*(1, 51) = 24.78, MSE = 871.93, *p* < 0.001, *η*^2^ = 0.33; main effect of orthography, *F*(1, 51) = 14.73, MSE = 894.91, *p* < 0.001, *η*^2^ = 0.22; mean peak latencies across conditions: 371–408 ms, SD: 40.46–44.04]. The N400 was present in both NH and HI (main effects of rhyme, *p*s < 0.019, *η*^2^ = 0.20 and 0.60, respectively) and peak latencies were shorter in the rhyming and orthographically similar conditions in both groups (main effects of rhyme and orthography, *p*s < 0.025, *η*^2^ = 0.20–0.30).

Like in long ISI, a left FP in response to phonological incongruence onset at around 400 ms and was significantly more positive in the non-rhyming than the rhyming conditions [*t*(104) = 2.96, *p* = 0.004, *r* = 0.28].

To summarize, a distinct N400 response was elicited by the paradigm but, contrary to our hypothesis, it did not differ between groups in either ISI.

#### 500–700 ms latency window

##### Long ISI

In this later time-window, waveforms showed a distinct left FP in response to incongruency and a continued N400 over the right centroparietal area (rhyme × orthography × antpos × hemisphere interaction). *Post hoc* tests showed that amplitudes over the left frontal area were significantly more positive in response to the non-rhyming than the rhyming conditions, and more positive in response to R−O− than R−O+ (*p*s < 0.001, *r*s = 0.76–0.94, mean amplitudes across conditions: −0.07 to 2.18 μV, SD: 1.84–2.16). The largest amplitude difference was measured at FC5 where mean amplitudes were −0.18 and 2.47 μV in R+O− and R−O−, respectively. The positivity was also sensitive to condition in terms of timing: peak latencies were longest in the R−O+ condition (*M* = 603 ms, SD = 51.04) and shortest in R+O+ (*M* = 586 ms, SD = 54.96), a difference that was significant [rhyme × orthography interaction, *F*(1, 51) = 5.45, MSE = 1842.73, *p* = 0.024; *post hoc* R+O+ versus R−O+ comparison, *t*(51) = 3.05, *p* = 0.004, *r* = 0.40].

In the right centroparietal area, amplitudes remained more negative in response to the non-rhyming than rhyming conditions (*p*s < 0.001, *r*s = 0.67–0.83) but no longer differentiated between R+O+ and R+O−. In contrast to the previous time-window, ERPs were most negative in response to R−O+ (R−O+ versus R−O−, *p* < 0.001, *r* = 0.47) suggesting a prolonged N400 effect to R−O+ target words. There were no group differences in ERP amplitudes or peak latencies in this time-window.

##### Short ISI

In short ISI, waveforms showed a similar, but less extended, positivity in response to incongruence over left frontal electrodes (rhyme × orthography × antpos × hemisphere interaction). As in long ISI, the positivity was larger in response to non-rhymes than rhymes, and in response to R−O− than R−O+ (mean amplitudes across conditions: −0.07 to 1.33 μV, SD: 1.82–2.07; *p*s < 0.001, *r*s = 0.48–0.90). Peak latencies were somewhat shorter than in long ISI, around 515 ms (R−O−, *M* = 512, SD = 43.62; R−O+, *M* = 518, SD = 46.65). ANOVA conducted on left frontal peak latencies resulted in a rhyme × orthography interaction [*F*(1, 51) = 10.04, MSE = 1096.53, *p* = 0.003]. Latencies were significantly longer in response to R−O− than the other conditions (*p*s < 0.001, *r*s = 0.39–0.54) but were not sensitive to group.

The N400, i.e., more negative amplitudes in response to non-rhymes than rhymes in the right centroparietal area, continued into this time-window (*p*s < 0.001, *r*s = 0.63–0.90). There was a significant rhyme × group interaction [*F*(1, 51) = 6.70, MSE = 2.91, *p* = 0.012] in ERP amplitudes: in NH participants ERPs no longer differentiated between rhymes and non-rhymes while there was a trend toward a continued differentiation in the hearing-impaired group [*t*(51) = 2.02, *p* = 0.048, *r* = 0.27, ns after Bonferroni correction].

The main finding in this time-window was that the rhyme judgment paradigm elicited a left FP, sensitive to phonology and showing an additive effect of phonological and orthographic incongruence in non-rhymes.

## Discussion

The present study investigated how HI affects the neural correlates of phonological processing. ERPs of participants with moderate-to-severe postlingually acquired HI and NH were registered during a visual rhyme judgment task with matching and mismatching orthography. The ISI was manipulated to examine behavioral performance and ERP correlates under conditions more or less conducive to employment of explicit processing. Behaviorally, the hearing-impaired participants performed worse than the NH participants in the mismatching rhyme judgment conditions when there was less time to engage top-down processes (short ISI). However, when such strategies could be more readily employed the hearing-impaired participants performed on a par with the NH participants (long ISI). Further, ERP results showed an amplified N2-like response, specific to the participants with HI and long ISI, in the rhyme judgment condition in which they benefited the most from long ISI.

### Behavioral results

Previous studies using similar material but with simultaneous presentation of the words in each word pair (Andersson and Lyxell, 1998; Lyxell et al., 1998; Andersson, 2002) have typically found that hearing-impaired participants fall behind participants with NH in visual rhyme judgments. It was therefore somewhat surprising that the groups performed on a par in long ISI in the present study. It is not unlikely that the hearing-impaired participants benefited from the explicit instruction to make decisions based on word phonology alone and the use of sequential, rather than simultaneous, presentation of the words in each pair in the present design. Fewer errors with sequential than simultaneous presentation, especially for non-rhyming pairs, have been reported previously (Johnston and McDermott, 1986). In addition, there was no difference between groups in non-word reading which indicates that our hearing-impaired participants had relatively good phonological decoding abilities. With this in mind, they still made significantly more errors than the NH participants in both mismatching conditions in short ISI, that is, when there was less time to deploy top-down processing to aid performance. They also performed significantly worse than the NH participants in the test of phonemically based verbal retrieval, results that are in line with the research indicating that auditory deprivation may be associated with degraded phonological representations. With long ISI and more time to engage top-down strategies, rhyme judgment performance between groups was equalized. It was also in long ISI that the hearing related enhanced N2-like negativity was found.

### ERP results

#### 100–300 ms latency window

N2-like negativities in response to phonological and/or orthographic processing in silent reading have been reported in several studies (Niznikiewicz and Squires, 1996; Proverbio and Zani, 2003; Newman and Connolly, 2004; Simon et al., 2004; Martin et al., 2006; Vissers et al., 2006) and are thought to reflect processes related to sublexical detection of conflict between outputs of orthographic/phonological analyses or violation of orthographic form expectations. The finding of an N2-like negativity sensitive to condition in the hearing-impaired group in the present study was in accordance with our hypothesis. Long-term acquired hearing loss in the moderate-to-severe range is known to affect phonological processing and compromise rhyme judgments when there is no support from orthography. Extra allocation of resources, reflected by an enhanced N2-like negativity, at detection of mismatching phonological and orthographic cues fit in under these circumstances. To our knowledge this is the first time such an effect has been reported in individuals with HI; as noted in the introduction, little attention has hitherto been given to investigating the effect of acquired hearing loss on the neural correlates of phonological processing. A similar N2-like effect has however recently been reported in individuals with dyslexia, another population which has been suggested to have specific problems with phonological representations and processing (Swan and Goswami, 1997; Elbro and Jensen, 2005; Ziegler and Goswami, 2005; Boada and Pennington, 2006). Savill and Thierry (2011) conducted a study where they used homophonic and non-homophonic pairs of non-word primes and word targets that were orthographically similar or dissimilar (e.g., the primes horce, hauce, horle, or hiele presented before the target word HORSE). The task was to decide whether prime and target sounded the same or not. Dyslexic participants showed significantly more negative potentials than controls in response to orthographically similar non-homophones (e.g., horle-horse, comparable to R−O+ in the present study) than to orthographically dissimilar non-homophones (e.g., hiele-horse, comparable to R−O−) in a 150- to 220-ms time-window. Thus, individuals with dyslexia and HI both show a neural sensitivity to orthographic similarity in phonologically different stimuli in visual phonological judgment tasks that is not found in individuals with intact phonological abilities.

In the present study, a phonological strategy was actively encouraged not just by the task itself, but also by the explicit instructions to make judgments based on how words sound and the consecutive (as compared to simultaneous) presentation of words within pairs. In rhyme judgments with long ISI, a phonological strategy leads to generation of an expectancy set involving lexical candidates that rhyme with the first word. Thereby, judgment of rhyming word pairs is facilitated (Hillinger, 1980; Johnston and McDermott, 1986) to a degree that can override the effect of conflicting orthography (Baum and Leonard, 1999; Savill et al., 2011). This is likely the reason why there was no N2-like enhancement in response to orthographically dissimilar rhymes, in spite of the presence of misleading orthographic cues. By contrast, in orthographically similar non-rhymes, phonological expectancies are always violated: the second word never rhymes with the first word. Hence, predictions have to be abandoned and rapid access made to the phonology of the second word. Indeed, several studies have found longer reaction times and/or lower accuracy in R−O+ than R+O− conditions (Polich et al., 1983; Johnston and McDermott, 1986; Kramer and Donchin, 1987; Rugg and Barrett, 1987; Lindell and Lum, 2008) indicating that the processing load generated by conflicting cues is largest when different phonological codes need to be derived from similar orthographic patterns (Weber-Fox et al., 2003).

We had expected that the poorer quality phonological representations associated with moderate-to-severe HI would interfere with implicit spreading of activation mechanisms as reflected by ERP deviations in short ISI, but this was not the case. Being aware of the problem with overlapping components in short ISIs (where responses elicited by a stimulus have not ended before a second stimulus is presented) and the concomitant risk of missing effects, it also needs to be noted that we chose to analyze correct answers only. In other words, our results showed no group difference in the neural correlates of *successful* identification of rhyme status in short ISI. Instead we found a pattern where behavioral performance benefited from long ISI specifically in the R−O+ condition, in which performance of the hearing-impaired group was on a par with the NH group in long (but not short) ISI, and a hearing related N2-like negativity enhanced only in R−O+ in long ISI. This pattern of results suggests the enhanced N2-like negativity in the hearing-impaired group is part of a compensatory strategy.

Functional Magnetic Resonance Imaging studies examining rhyme judgment in congenitally deaf and/or dyslexic adults have found increased activation in the left IFG, suggested to reflect compensatory use of spelling-to-sound procedures and fine-grained articulatory recoding (e.g., Aparicio et al., 2007; MacSweeney et al., 2009). The role of articulatory recoding in this task is also demonstrated by behavioral findings of the disruptive effect of articulatory suppression on visual rhyme judgment (Johnston and McDermott, 1986; Brown, 1987; Tree et al., 2011), particularly for orthographically similar non-rhyming word pairs (Johnston and McDermott, 1986). Thus, it is likely that in this study too, articulatory recoding and grapheme-to-phoneme conversion are examples of strategies used by the hearing-impaired participants to compensate for phonological processing difficulties. Such strategies would lead to an enhanced precision of the representation, orthographic, and/or phonological, of the first word. In turn, this would facilitate detection of mismatch at presentation of the second word.

In short ISI there was no time to employ such strategies, increasing the risk of being misled by the orthographic similarity. Indeed, the increased responsiveness to visual, rather than sound-based language cues found in individuals with acquired HI (Lee et al., 2001, 2007; Champoux et al., 2009) is likely to lead to a higher reliance on orthography in this text-based task. This would make the hearing-impaired participants more vulnerable to orthographic interference in both mismatching conditions in short ISIs, which is in line with the results presented here.

Parietal N2-negativities elicited by orthographic/phonological mismatch in silent reading paradigms are often left-lateralized (Niznikiewicz and Squires, 1996; Newman and Connolly, 2004; Simon et al., 2004). However, this is not always the case (Martin et al., 2006). Bearing in mind that the scalp distribution of ERPs is not necessarily a reliable indicator of underlying neuronal generators, the right lateralization of the N2-like effect in the hearing-impaired participants in the present study is interesting in view of recent fMRI data. Lazard et al. (2012) found right PSTG/SMG overactivation in individuals with postlingually acquired hearing loss during visual rhyme judgments. The present result is compatible with their hypothesis that auditory deprivation is followed by functional reorganization in the form of increased recruitment of right temporal areas in phonological processing. On a more speculative note, it has also been shown that orthographic processing is a right hemispheric strength. For example, visual half-field studies indicate that the right hemisphere is superior to the left hemisphere for orthographic matching tasks, and is able to make non-rhyme decisions based on orthographic analysis (for a review of right hemispheric language processing abilities, see Lindell, 2006). The distribution of the N2-like effect in the present study might thus be related to a stronger reliance on orthography in making the rhyme decision.

#### 300–500 ms latency window

Contrary to our hypothesis, we found no hearing related N400 deviations in either mean amplitude or peak latency. In view of the group interaction that emerged in the earlier time-window, the lack of N400 differences is not likely to be caused by lack of power. Further, an N400 was clearly elicited by the paradigm: in long ISI, the component differed between all four conditions, showing the same additive effect of phonological and orthographic incongruity as previously reported in the literature. The component distinguished between rhymes and non-rhymes, and between orthographically similar and dissimilar word pairs, in both groups. Hence, nor is the lack of group effects likely to be caused by factors related to the stimulus material. This is worth noticing: as far as we know, this is the first ERP study using written Swedish word pairs in a rhyme judgment task with matching and mismatching orthography.

Although there are problems with interpreting null effects, the present results indicate that while early, sublexical phonological level processes are affected by acquired HI, later, phonological/lexical level processes proceed normally. If, as suggested above, top-down processing promote early detection of phonological/orthographic incongruency, it is reasonable that there is no deviancy in the later N400 component which is driven precisely by incongruence. The result is also in line with previous research suggesting that phonological processing in individuals with postlingual auditory deprivation is less divergent than that of dyslexics (Lyxell et al., 2003). For comparison, Savill and Thierry (2011) found that early N2 anomalies in dyslexic participants were followed by ERP deviations well into later time-windows, including the P600 (Savill and Thierry, 2011).

#### 500–700 ms latency window

The N400 continued into this later time-window, particularly as elicited by orthographically similar non-rhymes. This prolonged N400 is consistent with the suggestion above that R−O+ is the most challenging rhyme condition. In this time-window there was a rhyme × group interaction in short ISI but as *post hoc* procedures showed there was no reliable effect of rhyme in either group we refrain from discussing it further.

A P600 was not elicited by the present paradigm; instead, ERPs showed a distinct left FP in response to non-rhymes, and particularly non-rhymes that were also orthographically dissimilar, in both groups and ISIs. This component is very similar to a FP reported in studies using sentences with more or less predictable endings (DeLong et al., 2011, 2012; Thornhill and Van Petten, 2012; Van Petten and Luka, 2012). The FP differs topographically from the P600 which is typically largest at centroparietal scalp sites. Further, there is now general consensus that the P600 is related to reprocessing cost, i.e., continued analysis or reanalysis of, for example, problematic sentences. The FP is as yet much less studied but has been suggested to reflect processes invoked when specific prediction of an upcoming word is disconfirmed (DeLong et al., 2012; Thornhill and Van Petten, 2012; Van Petten and Luka, 2012). Similar to the N400, the FP has been found to be smaller for highly predictable sentence endings than for less predictable sentence endings. Unlike the N400 its amplitude seems insensitive to the semantic similarity between an unexpected and highly expected sentence ending. Hence, while the sensitivity of the N400 extends to a conceptual level, the FP seems driven more exclusively by the appearance of an unpredictable word (Thornhill and Van Petten, 2012). The present observation of a FP is interesting; to our knowledge there are no previous reports of it being elicited in designs using word pairs and a phonological judgment task. However, our results are compatible with previous findings that the FP is sensitive to specific expectations at a lexical level (Thornhill and Van Petten, 2012).

## Conclusion

To summarize, the present results show an early ERP signature of acquired HI in visual rhyme judgment. The signature takes the shape of an N2-like negativity sensitive to orthography in non-rhymes. It was only elicited when there was time to recruit explicit processes and performance of the hearing-impaired participants was on a par with that of the NH. We therefore suggest it reflects compensatory top-down processing, probably via increased reliance on articulatory recoding and grapheme-to-phoneme conversion procedures. Such strategies likely enhances the precision of a phonological/orthographic representation held in working memory and facilitates detection of mismatch at an early, sublexical level at the stage of comparison to a second representation. The right parietal topography of the N2-like effect supports recruitment of right temporal areas for phonological processing following auditory deprivation. In the condition where there was not enough time for employment of top-down strategies, HI was associated with reduced rhyme judgment ability when phonology and orthography gave conflicting cues. This is suggested to reflect a higher reliance on visual, rather than sound-based, linguistic information and, thus, an increased vulnerability to orthographic interference. Taken together, these results provide further insight into the mechanisms by which engagement of explicit functions support language processing when audition is compromised.

## Conflict of Interest Statement

The authors declare that the research was conducted in the absence of any commercial or financial relationships that could be construed as a potential conflict of interest.

## Footnotes

^1^http://spraakbanken.gu.se

^2^http://www.pstnet.com/eprime.cfm

## Abbreviations

BestEarPTA, best ear pure tone average; EEG, electroencephalogram; ERP, event-related potential; fMRI, functional magnetic resonance imaging; FP, frontal positivity; HI, hearing impaired group; IFG, inferior frontal gyrus; ISI, interstimulus interval; NH, normal hearing group; O−, orthographically dissimilar; O+, orthographically similar; PSTG, posterior superior temporal gyrus; R−, non-rhyming; R+, rhyming; SMG, supramarginal gyrus.
