# Erratum: Early ERP signature of hearing impairment in visual rhyme judgment

**DOI:** 10.3389/fpsyg.2013.00897

**Published:** 2013-11-28

**Authors:** Elisabet Classon

**Affiliations:** Linnaeus Centre HEAD, The Swedish Institute for Disability Research, Department of Behavioural Sciences and Learning, Linköping UniversityLinköping, Sweden

**Keywords:** event-related potentials, hearing impairment, phonology, visual rhyme judgment, inter-stimulus interval, N2, N400, FP

This General Commentary provides a corrected version of Figure 6, page 10. The topographical map of the group with hearing impairment in the 100–300 ms time-window and R–O– condition has been corrected. Figure 6 shows the topographical distribution of the grand averaged ERPs in the normal hearing (NH) and hearing-impaired (HI) groups in the 100–300, 300–500, and 500–700 ms time-windows, long ISI. The rows show the distribution over the R+O+, R+O−, R−O+, and R−O− rhyme task conditions.

**Figure 6:**
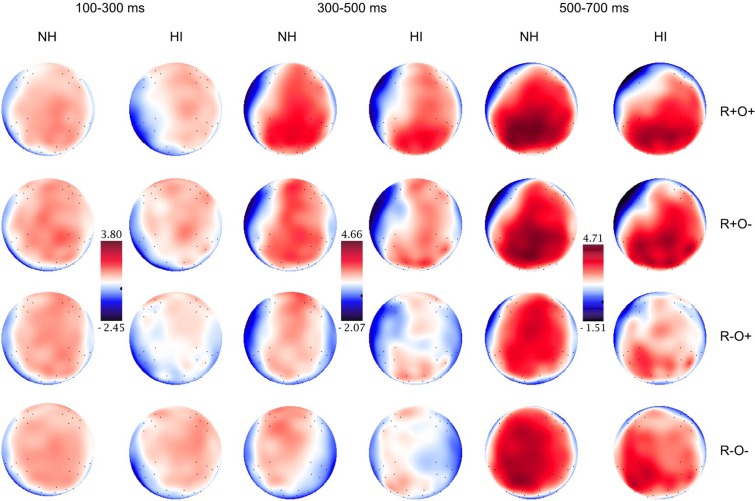
**Topographical distribution of the grand averaged ERPs in the normal hearing (NH) and hearing-impaired (HI) groups in the 100–300, 300–500, and 500–700 ms time-windows, long ISI.** The rows show the distribution over the R+O+, R+O−, R−O+, and R−O− rhyme task conditions.

